# New ligand-binding sites identified in the crystal structures of β-lactoglobulin complexes with desipramine

**DOI:** 10.1107/S2052252522004183

**Published:** 2022-04-29

**Authors:** Joanna I. Loch, Jakub Barciszewski, Joanna Śliwiak, Piotr Bonarek, Paulina Wróbel, Kinga Pokrywka, Ivan G. Shabalin, Wladek Minor, Mariusz Jaskolski, Krzysztof Lewiński

**Affiliations:** a Department of Crystal Chemistry and Crystal Physics, Faculty of Chemistry, Jagiellonian University, Kraków, Poland; b Institute of Bioorganic Chemistry, Polish Academy of Sciences, Poznan, Poland; c Department of Physical Biochemistry, Faculty of Biochemistry, Biophysics and Biotechnology, Jagiellonian University, Kraków, Poland; d Department of Molecular Physiology and Biological Physics, University of Virginia, Charlottesville, Virginia, USA; e Department of Crystallography, Faculty of Chemistry, A. Mickiewicz University, Poznan, Poland

**Keywords:** β-lactoglobulin, desipramine, ligand-binding sites

## Abstract

The homodimeric β-lactoglobulin belongs to the lipocalin family of proteins that transport a wide range of hydrophobic molecules and can be modified by mutagenesis to develop specificity for novel groups of ligands. In this work, new lactoglobulin variants, FAF (I56F/L39A/M107F) and FAW (I56F/L39A/M107W), were produced and their interactions with the tricyclic drug desipramine (DSM) were studied using X-ray crystallography, calorimetry (ITC) and circular dichroism (CD). The ITC and CD data showed micromolar affinity of the mutants for DSM and interactions according to the classical one-site binding model. However, the crystal structures unambiguously showed that the FAF and FAW dimers are capable of binding DSM not only inside the β-barrel as expected, but also at the dimer interface and at the entrance to the binding pocket. The presented high-resolution crystal structures therefore provide important evidence of the existence of alternative ligand-binding sites in the β-lactoglobulin molecule. Analysis of the crystal structures highlighted the importance of shape complementarity for ligand recognition and selectivity. The binding sites identified in the crystal structures of the FAF–DSM and FAW–DSM complexes together with data from the existing literature are used to establish a systematic classification of the ligand-binding sites in the β-lactoglobulin molecule.

## Abstract 2

New complexes of β-lactoglobulin with several ligand molecules simultaneously bound at different sites are presented and discussed in the context of a systematic classification of all possible binding sites in the lactoglobulin molecule.

## 1. Introduction

β-Lactoglobulin (BLG) is a homodimeric protein that naturally occurs in the milk of many mammalian species. Its physiological function is usually attributed as the binding and transport of fatty acids and fat-soluble vitamins. BLG can also interact with various drugs and therefore has been proposed as a potential carrier for many therapeutic agents (Gholami *et al.*, 2021 ▸; Ghalandari *et al.*, 2015 ▸; Balasco *et al.*, 2020 ▸; Kayani *et al.*, 2018 ▸). Like most lipocalins, the β-lactoglobulin molecule folds as an eight-stranded β-barrel, which is the primary ligand-binding site (Allahdad *et al.*, 2020 ▸; Dan *et al.*, 2019 ▸; Loch *et al.*, 2011 ▸, 2012 ▸, 2013 ▸, 2015 ▸, 2018 ▸; Sawyer, 2013 ▸; Patel *et al.*, 2019 ▸).

On the basis of spectroscopic, calorimetric and computational studies, several authors postulated that natural lactoglobulins possess additional (or secondary) binding sites located outside the β-barrel (Cho *et al.*, 1994 ▸; D’Alfonso *et al.*, 1999 ▸; Collini *et al.*, 2000 ▸, 2003 ▸; Domínguez-Ramírez *et al.*, 2013 ▸; Gholami & Bordbar, 2017 ▸; Al-Shabib *et al.*, 2018 ▸; Xu *et al.*, 2019 ▸). However, until now the only crystal structures of a lactoglobulin with a ligand bound outside the β-barrel were complexes of BLG with vitamin D_3_ (VD3; Yang *et al.*, 2008 ▸), tetracaine (Loch *et al.*, 2021 ▸) and SDS (Labra-Núñez *et al.*, 2021 ▸).

It has been shown that lipocalins can be re-engineered to gain new binding specificity (Gebauer & Skerra, 2020 ▸; Clifton *et al.*, 2019 ▸; Pelosi *et al.*, 2018 ▸; Ricatti *et al.*, 2019 ▸). This approach was also used by us to create a starting library of lactoglobulin mutants (Bonarek *et al.*, 2020 ▸). In further experiments, the starting library has been extended and new series of variants that possess multiple substitutions in the β-barrel were created. These variants had aromatic substitutions in the β-barrel in order to specifically recognize and bind drugs with tricyclic geometry. Systematic screening of ligand-binding preferences revealed some new BLG variants from our library with affinity for the tricyclic drug desipramine (DSM). This work presents part of our systematic studies of lactoglobulin–ligand interactions, focused on the binding of DSM to I56F/L39A/M107F (FAF) and I56F/L39A/M107W (FAW) mutants. The BLG I56F variant (Bonarek *et al.*, 2020 ▸) and other variants that contain this modification have a permanently reduced depth of the binding pocket. An aromatic residue at position 107 (phenylalanine or tryptophan) was introduced into the FAF and FAW variants to enhance the binding of ligands to aromatic fragments by creating π–π interactions. The L39A mutation enlarged the binding pocket in the AB loop region, creating space for the accommodation of larger ligands (Loch *et al.*, 2018 ▸).

Desipramine is on the list of toxic drugs that are intentionally overdosed by adults (Euwema & Swanson, 2019 ▸). Desipramine poisoning is difficult to treat because DSM is a lipophilic compound with a high distribution volume (O’Sullivan *et al.*, 2014 ▸). The design of new BLG variants capable of binding desipramine opens the possibility of their potential use in extracorporeal dialysis as part of protein-containing filtration membranes for removing toxic drugs. In this work, we present several high-resolution crystal structures of BLG–DSM complexes, providing unambiguous structural evidence for the existence of unique ligand-binding sites outside the β-barrel. The interactions of the new lactoglobulin variants with DSM were also confirmed by ITC calorimetry and circular dichroism.

## 2. Materials and methods

### 2.1. Mutagenesis, protein expression and purification

**Figure 1:**
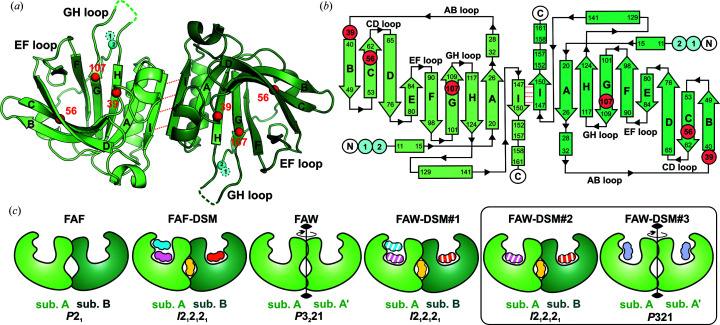
(*a*) Structure of the lactoglobulin homodimer with the mutation sites L39A, I56F and M107F (or M107W) marked by red spheres; cyan spheres mark the N-­terminal L1A/I2S substitutions. The dimer subunit *A* (chain *A*) is colored light green, while subunit *B* (chain *B*) is in dark green. (*b*) Topology diagram of the BLG homodimer with mutation sites marked as in (*a*) and with labels for β-strands (arrows) A–I and for the following strand-connecting loops: AB, CD, EF and GH. α-Helices are marked as rectangles without labels. In (*a*) and (*b*) the hydrogen bonds responsible for dimerization are marked by red dotted lines. (*c*) The green shapes represent BLG dimer subunits (light green, chain *A*; dark green, chain *B*). In FAF, FAF–DSM, FAW–DSM#1 and FAW–DSM#2 the asymmetric unit contains the BLG dimer (chain *A* and chain *B*), while in FAW and FAW–DSM#3 the dimer is generated by a crystallographic twofold axis (chain *A* and symmetry-related chain *A*′). The colored shapes represent DSM molecules: DSM I (pink), DSM II (cyan), DSM III (yellow), DSM IV (orange) and the single DSM in FAW–DSM#3 (blue). Ligands with fractional occupancy are marked with white stripes. Crystals of FAW–DSM#2 and FAW–DSM#3 grew simultaneously in the same crystallization drop (black frame). Space-group symbols are given at the bottom of (*c*).

Mutagenesis at position 107 (M107F or M107W; Fig. 1 ▸) used the coding sequence of the FA (I56F/L39A) variant cloned into pET-DUET-1 expression vector (Novagen; Loch *et al.*, 2018 ▸). Site-directed mutagenesis was performed using the QuikChange protocol. The presence of mutations was confirmed by DNA sequencing (Genomed S.A., Poland). The genes for both of the new variants, I56F/L39A/M107F (FAF) and I56F/L39A/M107W (FAW), carried the N-terminal L1A/I2S substitutions (Loch *et al.*, 2016 ▸). The FAF and FAW proteins were expressed and purified according to the previously published protocol #2 (Loch *et al.*, 2016 ▸). Native (natural) BLG was purchased from Merck and was purified by size-exclusion chromatography using a HiLoad 26/600 Superdex 75 column (GE Healthcare).

### 2.2. Crystallographic screening, crystallization, X-ray data collection and structure refinement

In the first steps of the project, the ligand affinities of the new lactoglobulin variants were screened by co-crystallization with a set of different molecules, including tricyclic drugs (for example desipramine, chlorpromazine and fluphenazine). Prior to the crystallization experiments, the proteins were concentrated to 22–33 mg ml^−1^. Screening was carried out with the use of 2.0–3.0 *M* ammonium sulfate (AS) in 0.5 *M* Tris buffer pH 8.5 and a 20-fold molar excess of ligands. The rapid (18–24 h) growth of relatively large crystals (up to 0.6–0.8 mm) usually indicated the formation of a BLG–ligand complex, and these crystals were used for X-ray data collection. In one of the screening trials using the FAF and FAW mutants, the growth of crystals with outstanding morphology (square-based elongated prisms; Supplementary Fig. S1) was observed in drops containing DSM. These crystals diffracted X-rays poorly (up to 2.5 Å resolution); however, the electron-density maps clearly indicated an unusual pattern of DSM binding. To obtain high-resolution data, further optimization of the crystallization conditions was performed using different protein and ligand concentrations, as well as different drop volumes.

Ultimately, the best-quality crystals of liganded and un­liganded FAF and FAW were obtained by the hanging-drop vapor-diffusion method using 2.2–2.4 *M* AS in 0.5 *M* Tris buffer pH 8.5. 2 µl protein solution was mixed with 2 µl precipitant solution and 0.5 µl DSM solution in water at an appropriate concentration. Diffraction-quality crystals of unliganded FAF and FAW grew in drops containing 2.4 *M* AS. Crystals of FAF and FAW complexes with DSM were obtained in drops containing a 20-fold molar excess of DSM using 2.4 *M* AS (FAF–DSM) or 2.2 *M* AS (FAW–DSM#1), respectively (Supplementary Table S1). A higher molar excess of DSM resulted in its heavy precipitation, which affected the growth of protein crystals. In one crystallization experiment the simultaneous growth of two crystal forms, FAF–DSM#2 and FAF–DSM#3, was observed in a drop containing 2.4 *M* AS and a 1:10 FAW:DSM molar ratio (Supplementary Table S1). The structures of the crystals originating from this experiment are also included in this work.

X-ray diffraction data were collected on the EMBL/DESY beamline P13 at PETRA III, Hamburg, Germany, beamlines 14.1 and 14.2 at BESSY II, Berlin, Germany and beamline 21-ID-F at the Advanced Photon Source (APS), Argonne, Illinois, USA. Data were processed using *XDS* (Kabsch, 2010 ▸) or *HKL*-3000 (Minor *et al.*, 2006 ▸). The structures were solved by molecular replacement with *Phaser* (McCoy *et al.*, 2007 ▸). The structures were refined using *REFMAC*5 (Murshudov *et al.*, 2011 ▸) or *Phenix* (Liebschner *et al.*, 2019 ▸) and the electron-density maps were inspected and the models corrected in *Coot* (Emsley *et al.*, 2010 ▸). Statistics of data collection and structure refinement are presented in Table 1 ▸. The final models and structure factors were deposited in the PDB, and the corresponding raw X-ray diffraction images were deposited in the MX-RDR or RepOD Raw Data Repositories (Table 1 ▸).

### 2.3. Circular dichroism (CD) and thermal stability

Near-UV CD spectra were recorded at room temperature on a Jasco J-710 spectropolarimeter with 10 mm path length using 30 µ*M* protein solution in 50 m*M* phosphate buffer pH 7.5. Titration was performed with a stock solution of 10 m*M* DSM in DMSO to reach a threefold molar excess of the drug. The final concentration of DMSO was 1%(*v*/*v*). Three scanning acquisitions were accumulated and averaged to yield the final spectrum and the spectra were corrected for buffer baseline. The induced CD (ICD) spectra of DSM in complex with protein were corrected by subtracting the spectrum of the protein solution recorded before ligand addition. The ellipticity was converted into a difference in the extinction coefficients for the protein spectra. The thermal stability of the FAW mutant was determined by two methods: nanoDSF using a Prometheus NT.48 instrument (NanoTemper Technologies GmbH) and circular dichroism (Jasco J-710). Experiments were carried out according to protocols described previously for the FAF variant (Loch *et al.*, 2018 ▸).

### 2.4. Isothermal titration calorimetry (ITC) 

ITC measurements were carried out with a Microcal iTC200 calorimeter (GE Healthcare) at 293 K. The reference power was set to 5 A, the stirring speed was 700 rev min^−1^ and a spacing of 150 s was used. Titrations of the FAF and FAW mutants, as well as the native form of the protein, were carried out in 25 m*M* HEPES buffer 7.5 containing 150 m*M* NaCl. In all experiments, the protein concentration (in the cell) was kept between 60 and 130 µ*M* (as determined by UV absorption at 280 nm) and the DSM concentration (in the syringe) was between 1 and 3 m*M*. The ligand was injected in 19 aliquots of 2 µl. Raw ITC data were analyzed with *Origin* 7.0 (OriginLab) to obtain the following thermodynamic parameters: stoichiometry (*N*), dissociation constant (*K*_d_) and the changes in enthalpy (Δ*H*_a_) and entropy (Δ*S*_a_). Data were measured in duplicate for validation. Blank experiments were also performed by titration of the ligand into the buffer. Since the integration of the peaks from the blank measurement resulted in comparable heat values, we decided to use *y*-translation of the data points obtained from the protein/DSM titration to avoid the accumulation of errors. Titrations were performed in Tris buffer pH 8.5, as in the crystallization condition; however, only titration of FAF resulted in measurable enthalpy.

## 3. Results

### 3.1. Overall fold and thermal stability of the BLG mutants

The CD spectra of FAF and FAW (Supplementary Fig. S2) were compared with the spectrum of the WT recombinant protein, which is identical to that of the natural (native) protein (Loch *et al.*, 2016 ▸). The FAF spectrum is consistent with the spectra recorded for other lactoglobulin mutants (Loch *et al.*, 2018 ▸), while for FAW a significant increase of the signal is observed in the entire spectrum range. The differential CD signal shown in the inset of Supplementary Fig. S2 indicates that the two minima at 293 and 286 nm derived from Trp19 and Trp61 are preserved but have a lower intensity. This difference can be explained by the presence of an additional tryptophan at position 107. The ratio of the absorption co­efficients ɛ_FAW_/ɛ_FAF_ is 1.32 and this value is consistent with the ratio of amplitudes at the 293 nm minimum. Therefore, it seems that Trp107 does not contribute significantly to the CD spectrum of FAW. The increase of the signal below 285 nm is unequivocal and may result from changes in the chiral environment of phenylalanine, tyrosine and S–S bridges.

To test how the introduction of Trp at position 107 affects protein stability, the thermal profile of FAW was monitored using CD and nanoDSF (Supplementary Fig. S2). Both methods gave very similar *T*_m_ values (72.3 ± 0.2 and 72.0 ± 0.1°C, respectively), which are very close to the *T*_m_ values of 71.7 or 71.3°C previously determined for FAF and indicate a slight decrease in the thermal stability of FAW in comparison to the WT protein (*T*_m_ = 79.7°C; Loch *et al.*, 2018 ▸).

### 3.2. Crystal structure of unliganded FAF and FAW

In the absence of a ligand, the FAF variant crystallized in space group *P*2_1_ with a protein dimer in the asymmetric unit. The overall fold of FAF is almost identical to that of WT lactoglobulin (PDB entry 6qi6; C^α^ r.m.s.d. of ∼0.3 Å). The phenylalanine residues introduced at positions 56 and 107 inside the binding pocket did not disrupt the conformation of the adjacent side chains (Supplementary Fig. S3). Chains *A* and *B* are almost identical except for small atomic shifts (up to 1.8 Å) observed mostly for the C^α^ atoms in the CD loop [see the topology diagram in Fig. 1 ▸(*b*)]. The conformation of the flexible EF loop (which acts as a gating element which regulates access to the β-barrel interior) can be classified as ‘open’, while the GH loop is disordered in both subunits, as is often observed in BLG crystals grown at a pH of about 8 (Qin *et al.*, 1998 ▸).

The binding pocket of FAF contains three phenylalanines at positions 56, 105 and 107 (Supplementary Fig. S3). The phenyl ring of Phe107 occupies a position in the center of the binding pocket and forms a T-shaped π–π interaction with Phe105 at the bottom of the pocket. The third phenylalanine is located on the side of the binding pocket and is surrounded by hydrophobic residues (mostly Leu). Such a combination of three aromatic rings in the binding pocket creates a strongly hydrophobic environment that is ready to accept ligands with aromatic fragments (Loch *et al.*, 2018 ▸).

Unliganded FAW, containing tryptophan at position 107, crystallized in space group *P*3_2_21 with one chain in the asymmetric unit and the typical symmetry of lactoglobulin crystals (Bonarek *et al.*, 2020 ▸). The overall fold of this mutant is very similar to that of the WT and FAF proteins (C^α^ r.m.s.d.s of 0.29 and 0.48 Å, respectively), the EF loop has an open conformation and the GH loop is partially disordered. The side chain of Trp107 is directed towards the entrance to the binding pocket and fills the space between the EF and GH loops. As a result, compared with FAF the entrance to the binding pocket is narrower but the pocket is deeper (Supplementary Fig. S3).

### 3.3. Crystal structure of the FAF–DSM complex

**Figure 2:**
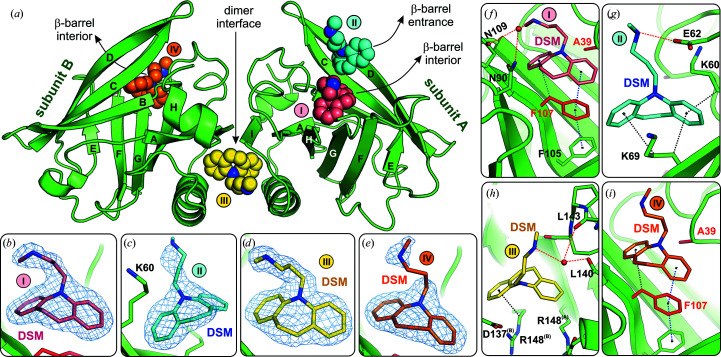
(*a*) Crystal structure of the FAF–DSM complex in cartoon representation with DSM molecules I, II, III and IV (space-filling representation) located at different binding sites (β-barrel interior, entrance and dimer interface). In subunit *A*, DSM I (pink) was bound in the β-barrel interior, DSM II (cyan) was found at the β-barrel entrance and DSM III (yellow) was located at the dimer interface. In subunit *B*, DSM IV (orange) was found in the β-barrel interior. (*b*)–(*e*) 2*F*_o_ − *F*_c_ electron-density maps around ligand molecules I, II, III and IV contoured at the 1.0σ level (difference omit maps are presented in Supplementary Fig. S1). (*f*)–(*i*) Interactions stabilizing the DSM molecules in their binding sites: hydrogen bonds (red dashed lines), hydrophobic contacts or C—H⋯π interactions (black dotted lines) and π–π stacking interactions (blue dotted lines). In (*h*), superscripts (A) or (B) mark residues belonging to chain *A* or *B*, respectively.

The FAF variant in the presence of DSM at a 1:20 molar ratio invariably crystallized in space group *I*2_1_2_1_2_1_ with the full dimer in the asymmetric unit. The open conformation of the EF loop regulating access to the β-barrel was slightly different from that in unliganded FAF, probably due to different crystal-packing contacts. The electron-density maps (Fig. 2 ▸) revealed that DSM is bound not only inside the β-barrel in both protein subunits (DSM I and IV in Fig. 2 ▸) but also at the dimer interface between the *A* and *B* chains (DSM III in Fig. 2 ▸). An additional DSM molecule was found at the entrance to the β-barrel of subunit *A* (DSM II in Fig. 2 ▸), while no ligand was identified in the same region of subunit *B*. The positions and occupancies of the DSM molecules in all structures are summarized in Supplementary Table S2 and Fig. 1 ▸(*c*).

The DSM molecules bound inside the β-barrel (DSM I and IV in Fig. 2 ▸) occupy almost the same position in both subunits and show a similar pattern of hydrophobic contacts. The tricyclic moiety of DSM matches the size and shape of the Phe107 side chain at the bottom of the binding pocket very well. One aromatic ring of the DSM molecule interacts with the Phe107 phenyl ring by π–π stacking, while another aromatic ring accepts a C—H⋯π hydrogen bond from the Phe107 C^β^ atom [Figs. 2( ▸ *f*) and 2 ▸(*i*)]. The aliphatic amine group of DSM molecule I forms water-mediated hydrogen bonds to the side chains of Asn90 and Asn109 [Fig. 2 ▸(*f*)].

The second DSM molecule bound to chain *A* (DSM II in Fig. 2 ▸) is located in the upper part of the β-barrel, at the entrance to the binding pocket, next to β-strands *C* and *D*. Its position is stabilized by C—H⋯π interactions between the ligand aromatic rings and the aliphatic segments of the Lys60 and Lys69 side chains, and additionally by the side chain of Leu87′ from the EF loop of a symmetry-related molecule. Additionally, the NH group in the DSM aliphatic chain forms a salt bridge to the carboxylate group of Glu62 [Fig. 2 ▸(*g*)]. No DSM molecule was found at an equivalent position in subunit *B*. This difference can be explained by the crystal packing. In subunit *A* this binding site is solvent-exposed, while in subunit *B* it is partially blocked by two other protein molecules. This indicates that binding inside the β-barrel preceded crystallization, while the additional DSM molecule II was bound when the protein was already in the crystalline state. This hypothesis can be confirmed by an analysis of ligand locations in the FAW–DSM#2 structure, which has identical unit-cell parameters and crystal packing (see Section 3.4). In FAW–DSM#2 the cleft responsible for DSM II binding is empty, so binding of the ligand at site II is not governed by crystal packing and symmetry.

Another DSM molecule was found at the dimer interface (DSM III in Fig. 2 ▸). It is bound in a cleft between the two subunits, surrounded by hydrophobic fragments of the side chains of Met145, His146 and Arg148 of chain *A* and Arg148, Phe138 and Leu133 of chain *B*. Comparative analysis of the crystal structures revealed that binding of the ligand at the dimer interface requires a rearrangement of Arg148 and Asp137 from chain *B* and local reorganization of the hydrogen-bond network (Supplementary Fig. S4). The binding at the dimer interface seems to result from shape and size complementarity between DSM and the cleft. This recognition is largely driven by hydrophobic forces. However, the DSM molecule is additionally stabilized at the dimer interface by water-mediated hydrogen bonds between the N2 atom of the DSM chain and the carbonyl groups of Leu140 and Leu143, and by a C—H⋯π interaction between the ligand aromatic ring and the aliphatic fragment of Arg148 [Fig. 2 ▸(*h*)].

### 3.4. Crystal structures of FAW–DSM complexes

**Figure 3:**
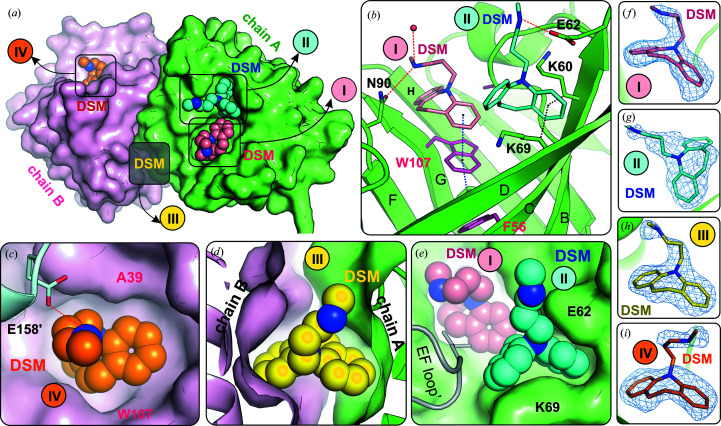
(*a*) Crystal structure of the FAW–DSM#1 complex. The molecular surface of the protein shows excellent shape complementarity between the ligands and the protein binding sites. DSM molecules I, II, III and IV (numbering as for FAF–DSM in Fig. 2 ▸) are shown in space-filling representation. (*b*) The interactions stabilizing DSM molecules I and II in the β-barrel sites of subunit *A*: hydrogen bonds (red dashed lines), hydrophobic contacts or C—H⋯π interactions (black dotted lines) and π–π stacking interactions (blue dotted lines). (*c*) DSM IV in subunit *B* is additionally stabilized by a hydrogen bond to Glu158′ from a symmetry-related molecule (marked by a prime). (*d*) Shape complementarity between DSM III and the binding site at the dimer interface (green, subunit *A*; pink, subunit *B*). (*e*) Shape of the binding sites in the β-barrel interior (DSM I) and β-barrel entrance (DSM II) of subunit *A*. DSM II at the β-barrel entrance is additionally stabilized by the EF loop from a symmetry-related FAW molecule. (*f*)–(*i*) 2*F*_o_ − *F*_c_ electron-density map contoured at 1.0σ around DSM ligand molecules I, II, III and IV (difference omit maps are presented in Supplementary Fig. S1).

**Figure 4:**
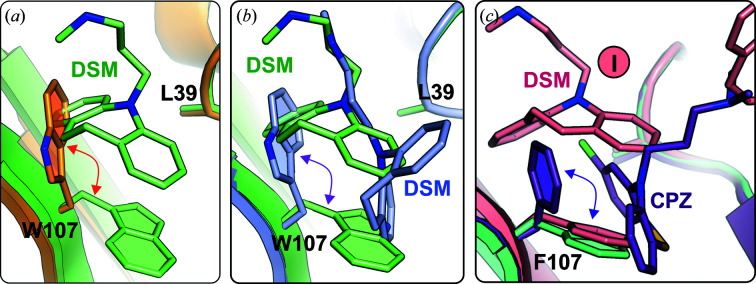
(*a*) Conformational changes of Trp107 associated with DSM binding in the β-barrel of the FAW–DSM#1 complex (green); the conformation of Trp107 in the unliganded protein is shown in orange. (*b*) Differences in the conformation of Trp107 and the position of DSM between FAW–DSM#1 (light green) and FAW–DSM#3 (light violet). (*c*) Conformational changes of Phe107 in FAF related to the ligand type accommodated in the β-barrel: Phe107 in unliganded FAF (light green), the conformation of Phe107 and the position of the ligand in the FAF–DSM complex (salmon) and the conformation of Phe107 and chlorpromazine in the FAF–CPZ complex (PDB entry 5nuk, violet).

Two different crystal forms of FAW in complex with DSM were obtained [Fig. 1 ▸(*c*)]. The FAW–DSM#1 complex crystallized from a solution with a 20-fold molar excess of DSM. The crystals have the same symmetry (*I*2_1_2_1_2_1_), unit-cell parameters and packing as those of the FAF–DSM complex (Table 1 ▸). The FAW dimer binds four DSM molecules at the same positions as in the FAF molecule (molecules I, II, III and IV in Fig. 3 ▸). The shape of the binding pocket in the β-barrel is different from that in unliganded FAW, but is almost identical to that in the FAF–DSM complex. This is the result of a conformational change of the Trp107 side chain, which is placed in the position occupied by the phenyl ring of Phe107 in FAF (Fig. 4 ▸). The conformational change of Trp107 facilitated the formation of π–π stacking interactions between the protein and ligand in the DSM–FAW#1 structure and also in the FAW–DSM#2 complex.

The refined occupancy of DSM molecules I, II and IV was approximately 0.9 (Supplementary Table S2). The DSM II ligand present at the entrance to the binding pocket of subunit *A* [Figs. 3 ▸(*b*) and 3 ▸(*e*)] has the same position and interaction pattern as in the equivalent molecule of the FAF–DSM structure [Fig. 2 ▸(*g*)]. Also, the position of the DSM III ligand located at the dimer interface is very well preserved in these structures [Figs. 2 ▸(*h*) and 3 ▸(*d*)]. The strong electron density indicates full occupancy of the ligand bound at this position [Fig. 3 ▸(*h*) and Supplementary Table S2]. The cleft at the dimer interface (as well as the β-barrel interior) that is responsible for anchoring the tricyclic fragment of DSM has hydrophobic character. The polar interactions (hydrogen bonds) at both sites that involved only the amino group at the aliphatic tail of DSM played a rather minor role in the binding.

In one of the crystallization trials, crystals with two different morphologies grew simultaneously in drops containing FAW and DSM in a 1:10 molar ratio (Supplementary Fig. S1). The first form (FAW–DSM#2) was isomorphous with the ortho­rhombic crystals of FAW–DSM#1 (and FAF–DSM), while the second form (FAW–DSM#3) was trigonal (*P*321) [Fig. 1 ▸(*c*), Supplementary Table S2].

In the FAW–DSM#2 structure [Fig. 1 ▸(*c*), Supplementary Fig. S5], strong electron density at the dimer interface clearly shows the presence of the DSM III molecule at full occupancy (Supplementary Table S2). The position of the ligand and its interactions are the same as in the FAW–DSM#1 structure. The primary binding site in the β-barrel is in both subunits has an occupancy of ∼0.8 (Supplementary Table S2). The electron density for DSM I and IV is weaker than in the FAW–DSM#1 structure and indicates positional and conformational disorder, in particular of Trp107 and DSM. The additional DSM II molecule that was observed at the entrance to the binding pocket of subunit *A* in FAW–DSM#1 could not be identified in FAW–DSM#2. There is a trace of the electron density near the CD loop, but it is too weak for reliable modeling of the ligand molecule.

The symmetry of the trigonal FAW–DSM#3 crystals [Fig. 1 ▸(*c*), Table 1 ▸], with one protein chain in the asymmetric unit, differs from the typical trigonal form of BLG–ligand complexes (Loch *et al.*, 2015 ▸, 2018 ▸; Bonarek *et al.*, 2020 ▸). The well defined electron density shows that the pH-sensitive flexible loops EF and GH have the open conformation and a single DSM molecule is bound inside the β-barrel [Figs. 1 ▸(*c*) and 5 ▸]. However, due to the position of the indole ring of Trp107, which is the same as in the unliganded form of FAW [Fig. 4 ▸(*b*) and Supplementary Fig. S3], the binding of the ligand molecule differs from that in other BLG–DSM complexes [Figs. 2 ▸(*f*) and 3 ▸(*b*)]. The DSM molecule is located deeper in the β­-barrel and one of its aromatic rings is held between a pair of C—H⋯π interactions provided by Val41 and Trp107 on both sides of the ring. The conformation of Trp107 in FAW–DSM#3, which is the same as in unliganded FAW, prevented the formation of π–π interactions between Trp107 and DSM bound in the β-barrel similar to those observed in the FAW–DSM#1 (and FAW–DSM#2) structure. As the trigonal form FAW–DSM#3 appeared only once in several crystallization trials, this observation suggests that the position of Trp107 forced less favorable interactions with the ligand and the orthorhombic form is preferred in the crystallization process.

**Figure 5:**
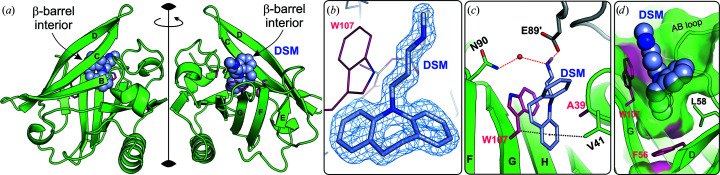
Structure of the FAF–DSM#3 complex. (*a*) The FAF dimer with crystallographically equivalent DSM binding sites inside the β-barrel. The crystallographic dyad is represented by a vertical black line. (*b*) 2*F*_o_ − *F*_c_ electron-density map (1.5σ) around the DSM molecule in the FAF–DSM#3 complex (a difference omit map is presented in Supplementary Fig. S1). (*c*) Interactions stabilizing the DSM molecule inside the β-barrel: hydrogen bonds (red dashed lines) and C—H⋯π interactions (black dashed lines). (*d*) The shape of the binding pocket and the pose of the DSM ligand in the β-­barrel.

In the crystal packing, the entrance to the β-barrel is blocked by the EF loop of another BLG molecule and the carboxyl group of Glu89′ from a symmetry-related molecule (denoted with a prime) forms a salt bridge with the NH group of the ligand (Fig. 5 ▸). The simultaneous growth of the trigonal and orthorhombic crystals of FAW when co-crystallized with DSM was observed in only one drop and could not be repeated. In other crystallization trials, only orthorhombic *I*2_1_2_1_2_1_ crystals (Table 1 ▸) grew.

### 3.5. Monitoring protein–ligand interactions by CD

Although DSM alone in the titration buffer gives no CD signal, an induced CD signal (ICD) appears after it has complexed with the protein (Supplementary Fig. S6). The ICD signal may be the result of molecular interactions between chiral (nonracemic) host and achiral guest compounds, and the rotational strength of the ICD band is proportional to the dipole strength of the electronic transition of the achiral molecule, the individual transition moments of the chiral molecules and their mutual geometric arrangement; it is inversely proportional to the quantities related to absorption frequency differences between the host and the guest (Allenmark, 2003 ▸). Our differential spectra (Supplementary Fig. S6, inset) revealed the presence of three additional bands centered at 255, 275 and 305 nm, consistent with the DSM absorption range (Sagdinc *et al.*, 2018 ▸). Analysis of the titration shows that the shape of the spectra is independent of the concentration of added DSM, suggesting the presence of only one binding site. The dissociation constants calculated according to the one-binding-site model are 65 ± 27 and 51 ± 20 µ*M* for FAF and FAW (Supplementary Fig. S7), respectively, which are in agreement with the ITC results (see Section 3.6). The stoichiometry for both variants is 1 (one DSM molecule per BLG chain). Since the binding constants determined for both variants are similar and the same reagent concentrations were used, similar concentrations of the complexes can be assumed for the data presented in the inset in Supplementary Fig. S6. Therefore, the difference between the ICD spectra is probably due to a different environment around the bound ligand. The positions of the extrema are generally preserved, but in the case of FAW an almost fourfold increase in their amplitudes is observed compared with FAF.

### 3.6. Monitoring protein–ligand interactions by ITC

ITC measurements of protein–ligand interactions at pH 8.5, at which the crystals of the complexes of FAF and FAW with DSM were obtained, were only successful for FAF, but with a very low enthalpy change (∼600 cal mol^−1^), while for FAW and the native protein the titrations resulted in an insufficient enthalpy change for the calculation of binding parameters. To compare the binding of DSM by both mutants and the native (natural) protein, the ITC measurements were repeated in HEPES buffer pH 7.5, in which the heat effect was strong enough to yield reliable binding parameters (Supplementary Fig. S8).

Titration of the FAF mutant with DSM at pH 8.5 or 7.5 resulted in *K*_d_ values of 39 ± 5 and 43 ± 6 µ*M*, respectively, which are comparable within experimental error. The stoichiometry calculated from fitting the data with a binding model was close to 1 at both pH values. The *K*_d_ value of 31 ± 6 µ*M* obtained for FAW at pH 7.5 indicates a slightly stronger binding of DSM than that of FAF, with an almost identical stoichiometry, while the natural protein binds DSM with a lower affinity than the mutants, with a *K*_d_ of ∼55 µ*M*. The stoichiometry and the thermodynamic parameters obtained from the ITC titrations are summarized in Table 2 ▸.

## 4. Discussion

### 4.1. Binding of tricyclic compounds by new β-lactoglobulin variants

In our studies of the interactions of FAF and FAW with DSM, the principal experimental method was X-ray crystallography. As β-lactoglobulin itself and the ligands used in the investigations (mostly hydrophobic drugs) do not exhibit specific biophysical properties (for example absorbance in the visible region) to facilitate simple, fast and affordable affinity screening, other approaches had to be used. Specifically, for the affinity screening of the new BLG variants, we used co-crystallization with a set of tricyclic ligands, one of which was desipramine. Co-crystallization of protein–ligand complexes allowed us to quickly detect positive hits (growth of crystals within 18–24 h), while initial home-source X-ray data collection enabled us to solve the structures and see the number of ligands complexed by the protein. In follow-up experiments, high-resolution crystal structures of DSM complexes were determined using synchrotron radiation (Table 1 ▸).

Although crystallography allows one to figure out the stoichiometry of binding (Fig. 1 ▸), it is of little use where the range of the binding constants, *K*_d_, is concerned. For this reason, our crystallographic studies were complemented by biophysical measurements such as CD and ITC. Due to the relatively high salt (ammonium sulfate) concentration, as well as the protein and ligand concentrations in the crystallization drop, the conditions used for crystal growth of the FAF–DSM and FAW–DSM complexes could not be directly used in the biophysical measurements. The buffers and the concentration conditions used in the CD and ITC experiments were a compromise between the conditions necessary to detect a specific binding effect and to reach an acceptable signal-to-noise ratio. Therefore, the protein concentration was selected based on preliminary experiments to prevent protein precipitation/aggregation upon titration with DSM, while the ligand concentration was adjusted according to its solubility in the titration buffer at a given pH.

The ITC experiments (Supplementary Fig. S8) and CD binding data (Supplementary Figs. S6 and S7) presented here indicated that both variants, FAF and FAW (as well as the native protein), bind approximately one ligand molecule per protein chain (Table 2 ▸). This result indicates that under the ITC experimental conditions DSM was preferentially bound inside the β-barrel of FAF and FAW, while the additional sites detected at the dimer interface and at the β-barrel entrance are probably available only when the concentrations of protein and ligand are very high (millimolar) and the equilibrium is shifted towards the dimeric form of BLG (Bonarek *et al.*, 2020 ▸; Mercadante *et al.*, 2012 ▸), for example in the crystalline phase. The affinity of the dimer interface and β-barrel entrance sites for DSM can be estimated from the crystallization conditions to be in the millimolar range (Supplementary Table S1). Interestingly, the natural protein binds DSM with a similar stoichiometry but a lower affinity compared with FAF or FAW (Supplementary Fig. S8 and S10). This observation suggests that DSM might also be accommodated in the β-barrel of the native protein; however, the binding is less specific and the ligand is probably located at the β-barrel entrance. The hydrophobic part of the binding pocket in the natural protein has an elongated shape [Supplementary Fig. S3(*a*)] and is too narrow to accommodate DSM. The lack of aromatic side chains at the β-barrel entrance probably prevents the formation of favorable π–π stacking interactions that could tightly stabilize DSM. Such a hypothesis should be confirmed by further structural studies; however, despite many trials, we were unable to crystallize a complex of the native protein with DSM. This observation indicates that natural BLG interacts with DSM in a different way to FAF and FAW.

In general, CD spectroscopy and microcalorimetry are global methods that do not directly locate the binding sites. The primary binding site located inside the BLG β-barrel usually has the highest ligand affinity, so changes in the CD signal and the enthalpic effects detected by ITC at pH 7.5 and 8.5 could be attributed with reasonable confidence to ligand binding inside the β-barrel of FAF and FAW. Thus, the crystal structures of the FAF and FAW complexes with DSM, showing binding of the ligand not only in the β-barrel but also at the dimer interface and at the β-barrel entrance near the CD loop, provide unique and important evidence of the presence of alternative ligand-binding sites in the BLG molecule. Only accurate high-resolution crystal structures allowed us to locate those sites with precision and confidence. The mutation sites 39, 56 and 107 are distant from the dimer interface, and they also did not affect the CD loop region or the conformation of the entire protein chain. In one crystallization experiment, we obtained an FAW–DSM#3 complex in which DSM was found only in the β-barrel. This result could not be repeated, and in subsequent crystallization trials only crystals similar to the FAW–DSM#1 form were obtained. These observations indicate that the FAF and FAW variants preferentially bind multiple ligand molecules per protein dimer.

The electron-density maps and refinement of the FAW–DSM#1 (and FAW–DSM#2) structure indicated that the binding sites in the β-barrel and at the dimer interface were fully (or almost fully) occupied by the DSM ligand in both complexes (Supplementary Table S2). The maps also showed that the binding site at the β-barrel entrance is almost fully saturated by DSM II in FAW–DSM#1, while in FAW–DSM#2 only a trace of the electron density corresponding to desipramine is visible. These observations indicate that the site at β-barrel entrance is less preferred and probably has a lower affinity for ligand than the other two sites.

In our previous report, we showed that the FA (I56F/L39A), FAF (I56F/L39A/M107F), LA (F105L/L39A) and LAF (F105L/L39A/M107F) mutants bind a single chlor­promazine (CPZ) molecule in the β-barrel (Loch *et al.*, 2018 ▸). The dissociation constants determined for the BLG–CPZ complexes were in the range 65–238 µ*M* (Loch *et al.*, 2018 ▸), while for DSM they are lower at 31–43 µ*M* (Table 1 ▸). In FAF–DSM, FAW–DSM#1 and FAW–DSM#2 the positions and orientations of the DSM I ligand in the β-barrel are the same, but are different from those observed in the FAF–CPZ complex (Fig. 4 ▸). This difference can be attributed to the conformational change of the Phe107 side chain (Supplementary Fig. S9), but also to the differences in the chemical structure between DSM and CPZ (Supplementary Fig. S11). As CPZ has a more rigid structure and a Cl atom attached to the tricyclic ring system, the accommodation of CPZ in the β-barrel required a conformational change of Phe107, which made room for the Cl atom in the cleft near Leu39.

Another significant difference between the complexes of FAF with CPZ and DSM is the presence of a DSM molecule in a cavity formed at the dimer interface. The BLG dimer has twofold symmetry, and two potential binding cavities are present at the dimer interface in the close vicinity of the Arg148 side chains from both subunits. Both cavities are occupied by the VD3 ligand in the BLG–VD3 complex (Supplementary Fig. S4). In contrast, in the FAF–DSM (and FAW–DSM#1 and FAW–DSM#2) complexes only one DSM molecule (DSM III) is bound close to the twofold axis located at the center of the dimer interface (Figs. 2 ▸ and 3 ▸). Analysis of these structures revealed that the accommodation of the single DSM III ligand was accompanied by a conformational change of the side chain of Arg148 in chain *B*, perturbing the local symmetry (Supplementary Fig. S4). In the FAF–DSM (and FAW–DSM#1 and FAW–DSM#2) complexes, the guanidinium moiety of Arg148 from chain *B* has shifted into the second cavity, rendering it inaccessible to the second ligand molecule. However, this conformational change of one Arg148 side chain created perfect room for the DSM molecule. The shape of the binding cavity at the dimer interface corresponds very well to the size and geometry of the DSM molecule [Fig. 3 ▸(*d*)]. Other tricyclic compounds, such as chlorpromazine, should also be able to bind at this site, but did not. As a possible explanation, the Cl atom of chlorpromazine is probably too large to fit at the dimer interface.

### 4.2. Ligand-binding sites in bovine β-lactoglobulin

**Figure 6:**
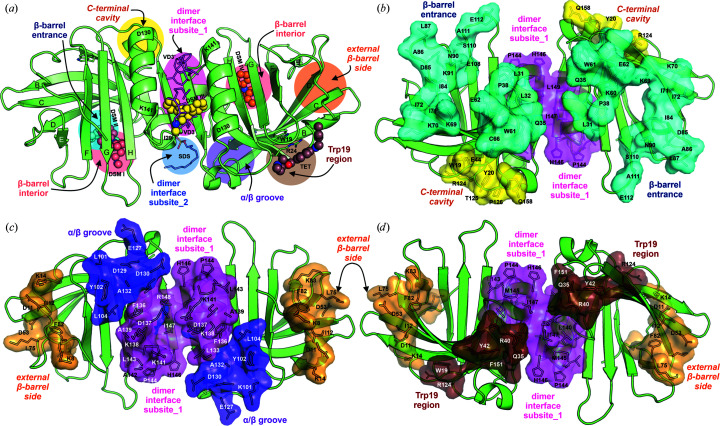
General classification of ligand-binding sites of the lactoglobulin dimer. (*a*) presents a superposition onto the BLG dimer frame of the ligands from the FAF–DSM structure, the BLG–VD3 complex (PDB entry 2gj5; VD3 and its symmetry-related copy VD3′), the I56–TET complex (PDB entry 7b8f) and the BLG–SDS complex (PDB entry 7kp5). (*b*), (*c*) and (*d*) present ligand-binding sites located outside the β-barrel (for the BLG molecule shown in different orientations). These sites are mapped onto the BLG dimer structure, with a detailed description given in Supplementary Table S3. Color codes: salmon, β-barrel interior, primary site; yellow, site at the C-terminal cavity; cyan, β-barrel entrance; violet, α/β groove; brown, Trp19 region; orange, external β-barrel side; magenta, dimer interface, subsite_1; blue, dimer interface, subsite_2. Subsite_2 partially overlaps with the Trp19 region (brown) and α/β groove (violet) and was omitted from (*b*), (*c*) and (*d*) for clarity.

Although the vast majority of ligands bind to β-lacto­globulin inside the β-barrel, the presence of additional binding sites has been postulated by several authors. Supplementary Table S3 and Fig. 6 ▸ show the sites identified in the crystal structures and NMR experiments and those proposed on the basis of spectroscopic studies combined with molecular modeling. Results based solely on modeling have been omitted. We classified the binding sites into six regions, although in some cases their distinction is arbitrary (Supplementary Table S3). The projection of the proposed binding sites onto the protein surface (Fig. 6 ▸) shows that almost all of its regions should be able to bind ligands. Some of these sites seem to be more selective and bind only ligands of specific structure, while others, for example at the β-barrel entrance, can bind a wide variety of compounds.

More than 200 different compounds have been reported to bind to β-lactoglobulin (Sawyer, 2013 ▸). For some of them, spectroscopic studies indicated the presence of ligands outside the primary binding site in the β-barrel, and molecular docking allowed an alternative binding region to be proposed. Most of these binding sites were located in the region near Trp19 or at the β-barrel entrance. As shown by the crystal structures of DSM complexes of FAW and FAF, the site at the β-barrel entrance is independent of the primary binding site inside the β-barrel. It may be occupied not only by DSM but also by other organic compounds such as 8-anilinonaphthalene-1-sulfonic acid (ANS; Collini *et al.*, 2003 ▸) or doxorubicin (Agudelo *et al.*, 2012 ▸).

### 4.3. Factors affecting selection of BLG ligand-binding sites

Ligand binding to β-lactoglobulin and the availability of binding sites depend on several factors. The most widely studied Tanford transition (Tanford *et al.*, 1959 ▸) relates the accessibility of the primary binding site inside the β-barrel to pH-dependent conformational changes of certain gating loops. In general, at a pH below ∼6.5 the EF and GH loops are in the closed conformation and block access to the binding pocket inside the β-barrel (Sakurai & Goto, 2006 ▸). When access to the pocket is limited, ligands may prefer surface binding sites. However, due to the flexibility of the EF and GH loops and the equilibrium between open and closed conformations of the loops, binding of ligands in the β-barrel at a lower pH, for example 4.5, is also possible (Labra-Núñez *et al.*, 2021 ▸). The pH also affects the oligomeric state of BLG. At pH values below 2.6 (Mercadante *et al.*, 2012 ▸), when BLG dissociates into monomers, additional ligand-binding regions become accessible on the protein surface, while the sites formed at the dimer interface will obviously disappear (Stender *et al.*, 2019 ▸; Birch *et al.*, 2021 ▸).

It has been shown that even small changes in the protein sequence can affect the number of ligand molecules that interact with BLG. For example, modification of Ile56, which is located within the β-barrel, changed the binding of tetracaine. Natural lactoglobulin accommodated a tetracaine molecule in the β-barrel (Loch *et al.*, 2015 ▸), while the I56F variant only binds the ligand on the protein surface (Loch *et al.*, 2021 ▸). The genetic variants of milk BLG, isoforms A, B and C, have, for example, different numbers of binding sites for retinol and epigallocatechin gallate (Keppler *et al.*, 2014 ▸). These observations indicate that even one or two substitutions, located far away from each other, can affect the number of ligand-binding sites, which is consistent with the results presented in this paper.

The results of the structural studies presented in this work, together with our previous observations, clearly indicate that ligand binding by lactoglobulin is mainly driven by nonspecific hydrophobic interactions. Therefore, shape complementarity is a major factor in ligand recognition, as can be seen for the DSM molecules in the β-barrel and at the dimer interface (Figs. 3 ▸ and 5 ▸). Although polar interactions (hydrogen bonds) with water molecules and protein residues can be found in complexes of BLG with DSM (Figs. 2 ▸ and 3 ▸) and many other ligands, our previous studies have shown that they play a secondary role in ligand binding in the β-barrel (Loch *et al.*, 2013 ▸). This makes docking simulations unreliable if they is not supported by additional physicochemical data.

Analysis of the binding sites on the BLG surface (Supplementary Table S3, Fig. 6 ▸) shows that some of them were identified close to the dimer interface. In monomeric BLG binding of ligands at these sites may be possible, but their availability may be severely limited in the dimeric form, which is the physiological state of BLG. It was also previously demonstrated that ligand binding can affect the equilibrium between the monomeric and dimeric fractions of lactoglobulin (Gutiérrez-Magdaleno *et al.*, 2013 ▸). The structures of BLG in complex with VD3 and DSM provide evidence that there are also genuine ligand-binding sites at the dimer interface, as they require the participation of both subunits. The formation of dimers also affects the distribution of the electrostatic field on the protein surface. Therefore, *in silico* ligand-binding studies should always be carried out for dimeric BLG.

## . Conclusions

Natural β-lactoglobulin is the prototypical dimeric lipocalin, with affinity for a wide range of ligands. Using site-directed mutagenesis, we designed and produced β-lactoglobulin FAF and FAW variants with the primary binding site in the β-barrel modified to accommodate tricyclic compounds. The affinity of FAF and FAW for desipramine and the crystal structures of the corresponding complexes were determined. Unexpectedly, the crystal structures revealed that DSM is present not only inside the β-barrel but also at the β-barrel entrance and, most importantly, in a cavity at the dimer interface. The crystal structures therefore provide experimental evidence that the BLG dimer can accommodate several ligand molecules simultaneously at different binding sites. The determined *K*_d_ values (micromolar) indicate that the site in the β-barrel interior has the highest affinity for DSM, while the affinity of the other sites is much lower, probably in the millimolar range. The structures presented in this paper also indicate the important role of shape complementarity of the ligand-binding site. Therefore, the presented study can be considered as the first step towards the production of modified lacto­globulin molecules that can act as specialized drug carriers or toxin scavengers capable of binding various low-molecular-weight ligands of biological and medical importance with high affinity and specificity.

## 6. Related literature

The following references are cited in the supporting information for this article: Ghalandari *et al.* (2014 ▸), Liu *et al.* (2017 ▸), Lübke *et al.* (2002 ▸), Maity *et al.* (2016 ▸) and Pantusa *et al.* (2014 ▸).

## Supplementary Material

PDB reference: FAF, 7q18

PDB reference: FAF–DSM, 7q2n

PDB reference: FAW, 7q17

PDB reference: FAW–DSM#1, 7q2o

PDB reference: FAW–DSM#2, 7q2p

PDB reference: FAW–DSM#3, 7q19

Raw diffraction images for FAF.: https://doi.org/10.18150/3BXUVE

Raw diffraction images for FAF-DSM.: https://doi.org/10.18150/RTXPKH

Raw diffraction images for FAW.: https://doi.org/10.18150/V2BG7N

Raw diffraction images for FAW-DSM#1.: https://doi.org/10.18150/C8ZOOT

Raw diffraction images for FAW-DSM#2.: https://doi.org/10.18150/2ABUX

Raw diffraction images for FAW-DSM#3.: https://doi.org/10.18150/SSQN7W

Supplementary Tables and Figures. DOI: 10.1107/S2052252522004183/lz5055sup1.pdf

## Figures and Tables

**Table 1:** Data-collection and structure-refinement statistics Values in parentheses are for the highest resolution shell.

BLG mutant	I56F/L39A/M107F (FAF)		I56F/L39A/M107W (FAW)			
Structure	FAF	FAF–DSM	FAW	FAW–DSM#1	FAW–DSM#2	FAW–DSM#3
Data processing						
Beamline	21-ID-F, APS	EMBL P13, PETRA III	14.2, BESSY II	14.2, BESSY II	14.1, BESSY II	14.1, BESSY II
Wavelength (Å)	0.97872	0.97625	0.91840	0.91840	0.91840	0.91840
Space group	*P*2_1_	*I*2_1_2_1_2_1_	*P*3_2_21	*I*2_1_2_1_2_1_	*I*2_1_2_1_2_1_	*P*321
*a*, *b*, *c* (Å)	45.91, 64.30, 55.50	55.82, 73.34, 179.70	53.08, 53.08, 111.47	55.67, 70.15, 179.02	55.81, 70.52, 178.80	66.30, 66.30, 60.63
α, β, γ (°)	90, 112.89, 90	90, 90, 90	90, 90, 90	90, 90, 90	90, 90, 90	90, 90, 90
Resolution limit (Å)	50.00–1.80 (1.83–1.80)	46.38–1.70 (1.80–1.70)	45.97–1.80 (1.91–1.80)	45.45–1.80 (1.91–1.80)	45.52–1.69 (1.79–1.69)	41.69–1.55 (1.64–1.55)
Total No. of reflections	101450	529706	139531	181280	517429	445871
No. of unique reflections	27108 (1363)	41144 (6512)	17450 (2615)	32842 (5196)	39985 (6354)	22796 (3482)
Multiplicity	3.70 (3.70)	12.90 (12.70)	4.31 (4.02)	5.52 (5.60)	12.94 (12.6)	10.30 (9.11)
〈*I*/σ(*I*)〉	35.10 (2.00)	17.97 (1.88)	13.73 (1.30)	14.42 (1.63)	19.43 (1.63)	23.24 (1.98)
*R*_meas_ (%)	8.0 (45.4)	6.9 (135.1)	5.7 (97.2)	7.4 (119.1)	7.2 (168.0)	5.1 (102.2)
Completeness (%)	99.0 (98.5)	99.8 (98.6)	99.8 (99.1)	99.2 (98.7)	99.6 (99.3)	99.9 (99.4)
CC_1/2_ (%)	99.2 (88.4)	100.0 (90.3)	99.9 (59.0)	99.9 (85.1)	100.0 (85.7)	100.0 (87.0)
Structure refinement						
Reflections (total/test set)	26016/1074	40027/1042	16448/1000	31839/1000	38980/1000	21796/1000
*R*/*R*_free_ (%)	19.9/23.3	20.4/23.3	18.3/22.6	18.4/22.1	18.9/23.6	19.9/23.4
R.m.s.d., bond lengths (Å)	0.011	0.010	0.011	0.011	0.012	0.011
R.m.s.d., angles (°)	1.66	1.60	1.62	1.68	1.68	1.66
Ramachandran plot statistics (%)						
Allowed	96	98	97	98	98	98
Favored	4	2	3	2	2	2
Outliers	0	0	0	0	0	0
PDB code	7q18	7q2n	7q17	7q2o	7q2p	7q19
Raw diffraction data	https://doi.org/10.18150/3BXUVE	https://doi.org/10.18150/RTXPKH	https://doi.org/10.18150/V2BG7N	https://doi.org/10.18150/C8ZOOT	https://doi.org/10.18150/2ABUX2	https://doi.org/10.18150/SSQN7W

**Table 2:** Thermodynamic parameters obtained from ITC titration of natural β-­lactoglobulin and its mutants with DSM at 293 K *N*, stoichiometry; *K*_d_, dissociation constant; Δ*H*_a_, enthalpy change; Δ*S*_a_, entropy change. The errors shown are the errors of fitting the data to the one set of binding sites model.

	Natural (pH 7.5)	FAF (pH 7.5)	FAW (pH 7.5)	FAF (pH 8.5)
*N*	0.91 ± 0.08	0.94 ± 0.06	0.92 ± 0.05	1.21 ± 0.08
*K*_d_ (µ*M*)	55 ± 9	43 ± 6	31 ± 6	39 ± 5
Δ*H*_a_ (cal mol^−1^)	−1298 ± 145	−1634 ± 133	−1792 ± 137	−614 ± 53
Δ*S*_a_ (cal mol^−1^ K^−1^)	15.0	14.4	14.5	18.1
*T*Δ*S*_a_ (cal mol^−1^)	4395.0	4219.2	4248.5	5303.3
