# Supplementary material for: New ligand-binding sites identified in the crystal structures of β-lactoglobulin complexes with desipramine
Source: IUCrJ. 2022 Apr 29;9(Pt 3):386–98. doi: 10.1107/S2052252522004183 (PMC9067113; doi:10.1107/S2052252522004183)
Supplement: Supplementary file 1 [file m-09-00386-sup1.pdf]

IUCrJ

Volume 9 (2022)

Supporting information for article:

New ligand-binding sites identified in the crystal structures of β-lactoglobulin complexes with desipramine

Joanna I. Loch, Jakub Barciszewski, Joanna Śliwiak, Piotr Bonarek, Paulina Wróbel, Kinga Pokrywka, Ivan G. Shabalin, Wladek Minor, Mariusz Jaskolski and Krzysztof Lewiński

Supplementary Tables

Table S1. Composition of the crystallization drops (AS = ammonium sulfate).

	protein concentration per monomer [mM]	DSM concentration [mM]	molar ratio protein (monomer):DSM	molar ratio protein (dimer):DSM	condition of crystal growth
**FAF-DSM**	0.54	11.0	~1:20	~1:40	2.4 M AS, 0.5 M Tris pH 8.5
**FAW-DSM#1**	0.54	11.0	~1:20	~1:40	2.2 M AS, 0.5 M Tris pH 8.5
**FAW-DSM#2**	0.98	9.1	~1:10	~1:20	2.4 M AS, 0.5 M Tris pH 8.5
**FAW-DSM#3**					

Table S2. Summary of ligand occupancies in the presented crystal structures.

**structure**		**FAF**	**FAF-DSM**	**FAW**	**FAW-DSM#1**	**FAW-DSM#2**	**FAW-DSM#3**
**space group symmetry**		$P2_1$	$I2_12_12_1$	$P3_221$	$I2_12_12_1$	$I2_12_12_1$	$P321$
**ASU contents**		dimer	dimer	½ dimer	dimer	dimer	½ dimer
**ligand in**	**β-barrel**	no	full occupancy	no	occupancy ~0.90	disorder/occupancy ~0.8	full occupancy
	**β-barrel entrance**	no	full occupancy	no	occupancy ~0.90	no	no
	**dimer interface**	no	full occupancy	no	full occupancy	full occupancy	no

Table S3. Classification of the ligand binding sites of the lactoglobulin molecule. Primed residues are from the complementary subunit of the BLG dimer. The primary binding site located in the β-barrel interior has been omitted in this classification, as it was described in detail several times for different types of ligands.

No.	Ligand	Residues	Method	Reference
dimer interface				
subsite_1 (cleft between α-helices 130-141)				
1	vitamin D3	F136-L149	X-ray	(Yang *et al.*, 2008)
2	desipramine	M145, H146, R148, L133’, F138’, R148’	X-ray	this work
subsite_2 (region between AB-loops)				
3	SDS	I29, S30, D33, A34’, Q35’, R40’, Q155’, H161’	X-ray	(Labra-Núñez *et al.*, 2021)
Trp19 region				
4	resveratrol	W19, R124	spectroscopy, docking	(Pantusa *et al.*, 2014)
5	rhein	W19, S21, Q35, R40, Y42	spectroscopy, docking	(Xu *et al.*, 2019)
6	emodin	W19	spectroscopy, docking	(Xu *et al.*, 2019)
7	chrysophanol	W19, S21, F151	spectroscopy, docking	(Xu *et al.*, 2019)
8	tetracaine	W19, R124	X-ray	(Loch *et al.*, 2021)
β-barrel entrance				
9	ANS	K60, K69	spectroscopy, docking	(Collini *et al.*, 2003)
10	doxorubicin	E62, K69, K70, I71, I72, I84, D85, A86, N90	spectroscopy, docking	(Agudelo *et al.*, 2012)
11	N-(trifluoroacetyl) doxorubicin	L31, L32, P38, V41, K60, E62, K69, I71, I84	spectroscopy, docking	(Agudelo *et al.*, 2012)
12	oxali-palladium	Q35, W61, C66	spectroscopy, docking	(Ghalandari *et al.*, 2014)
13	rutin	L1, K91, E108, S110, A111, E112	spectroscopy, docking	(Al-Shabib *et al.*, 2018)
14	desipramine	K60, K69, E62	X-ray	this work
15	exopolysaccharides	K70, I71, I72, L87	NMR at pH 2.65	(Birch *et al.*, 2021)
16	SDS	Q35, S30’, A33’	X-ray	(Labra-Núñez *et al.*, 2021)
C-terminal cavity				
17	isoxazole curcumin derivative	W19, Y20, E44, R124, E158	spectroscopy, docking	(Maity *et al.*, 2016)
18	curcumin	W19, Y20, E44, T125, P126, E158	spectroscopy, docking	(Liu *et al.*, 2017)
α/β groove				
19	β-ionone	Y102, L104, D129	NMR at pH 2.00	(Lübke *et al.*, 2002)
20*	exopolysaccharides	K101, Y102, D129	NMR at pH 2.65	(Birch *et al.*, 2021)
21*	alginate oligosaccharides	K101, E127, D129, D130, A132, D137	NMR at pH 2.65	(Stender *et al.*, 2019)
external β-barrel side				
22	alginate oligosaccharides	D11, E12, K14, K75	NMR at pH 2.65	(Stender *et al.*, 2019)
23	alginate oligosaccharides	K8, D11, E12, D53, K75, A80, F82, K83	NMR at pH 4.00	(Stender *et al.*, 2019)

* sites available in monomeric protein as described by the authors of the cited paper

Supplementary figures

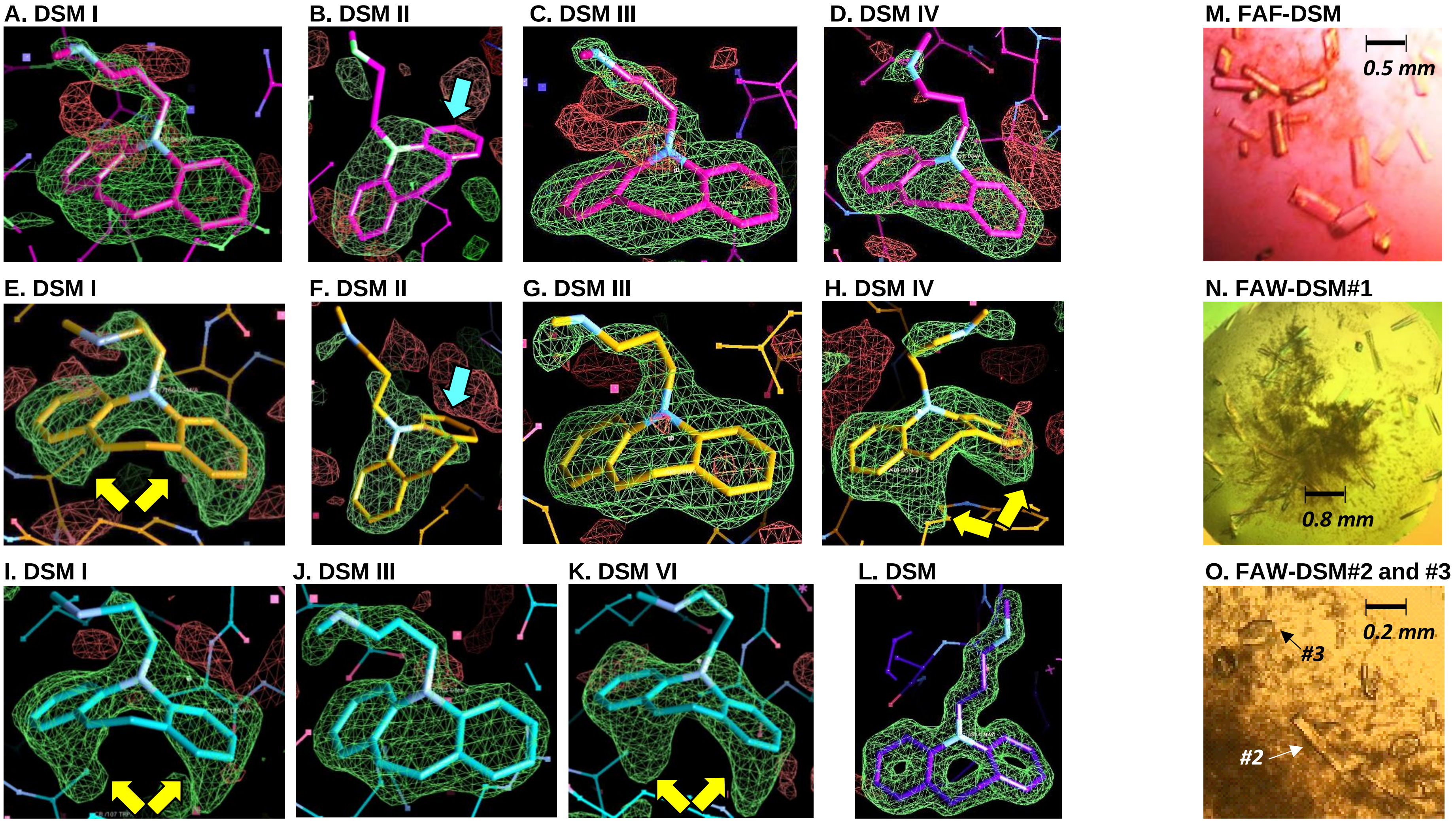

Fig. S1. Collection of Fo-Fc difference omit maps (all at 3.0σ level) for the ligand molecules located in the FAF and FAW complexes with DSM. (A-D) Ligands DSM I, II, III and IV in the FAF-DSM complex. (E-H) Ligands DSM I, II, III and IV in the FAW-DSM#1 complex. (I-K) Ligands DSM I, III and IV (DSM II is absent) in the FAW-DSM#2 complex. (L) The single DSM molecule in the FAW-DSM#3 complex. In panels A-L cyan arrows point to a fragment of the DSM II molecule that is exposed to solvent and has the weakest electron density, not visible at the 3.0σ level. Yellow arrows mark fragments of elongated electron density blobs visible near the DSM ligand in the β-barrel as the effect of alternative conformation of Trp107 and DSM at this position. As the refined occupancy of the alternative conformers does not exceed 10% in FAW-DSM#1 and 20% in FAW-DSM#2 (Table S3), they were not included in the final model. Panels (M-O) show typical morphology of the crystals of the FAF-DSM and FAW-DSM complexes.

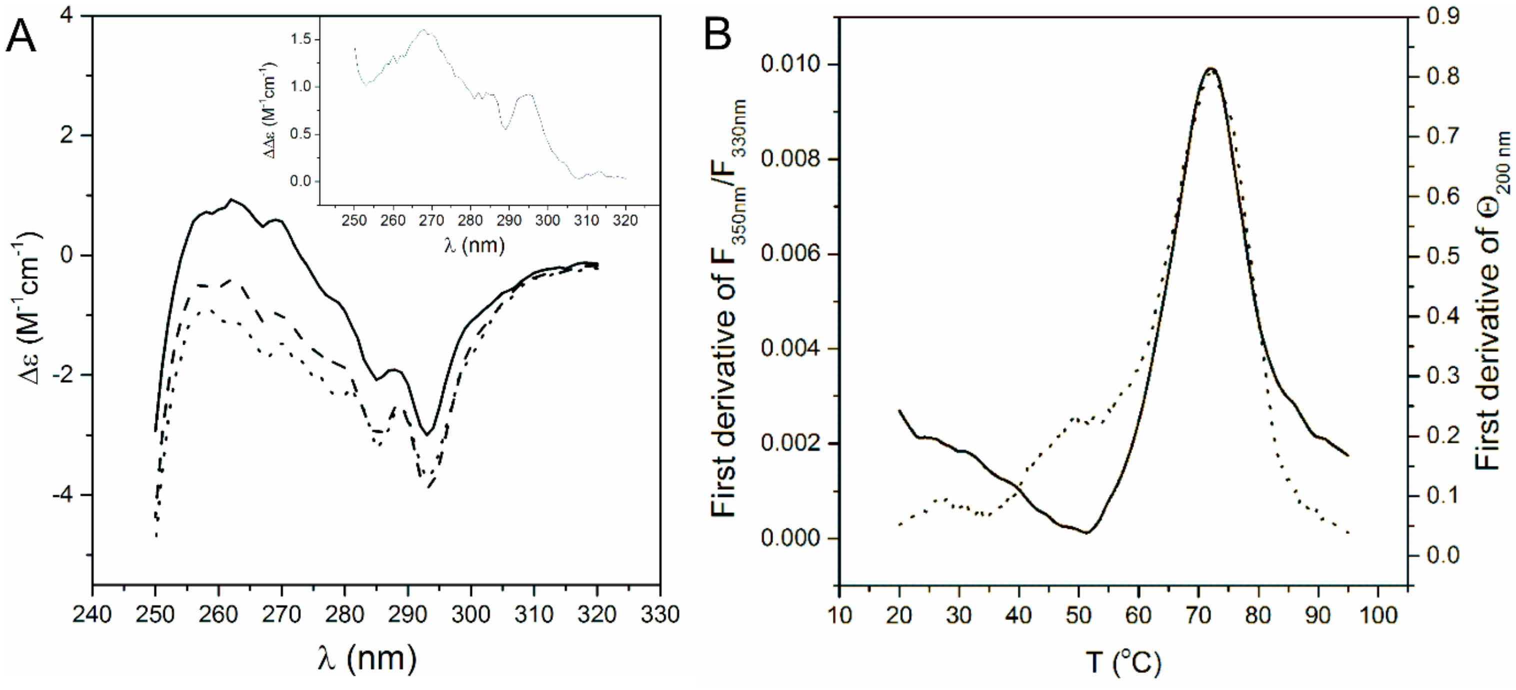

Fig. S2. (A) Near-UV CD spectra of FAW (solid line) and FAF (dashed line) in 50 mM phosphate buffer pH 7.5 at room temperature. The spectrum of WT from (Loch *et al.*, 2016) was added for comparison (dotted line). The inset presents the differential spectrum between FAW and FAF. (B) Thermal denaturation measured by nanoDSF (solid line) and CD (dotted line) of FAW in 50 mM phosphate buffer pH 6.5 with 1°C/min heating ramp. The data are presented as the first derivative of the ellipticity for 200 nm or the ratio of fluorescence intensity measured at 350 and 330 nm.

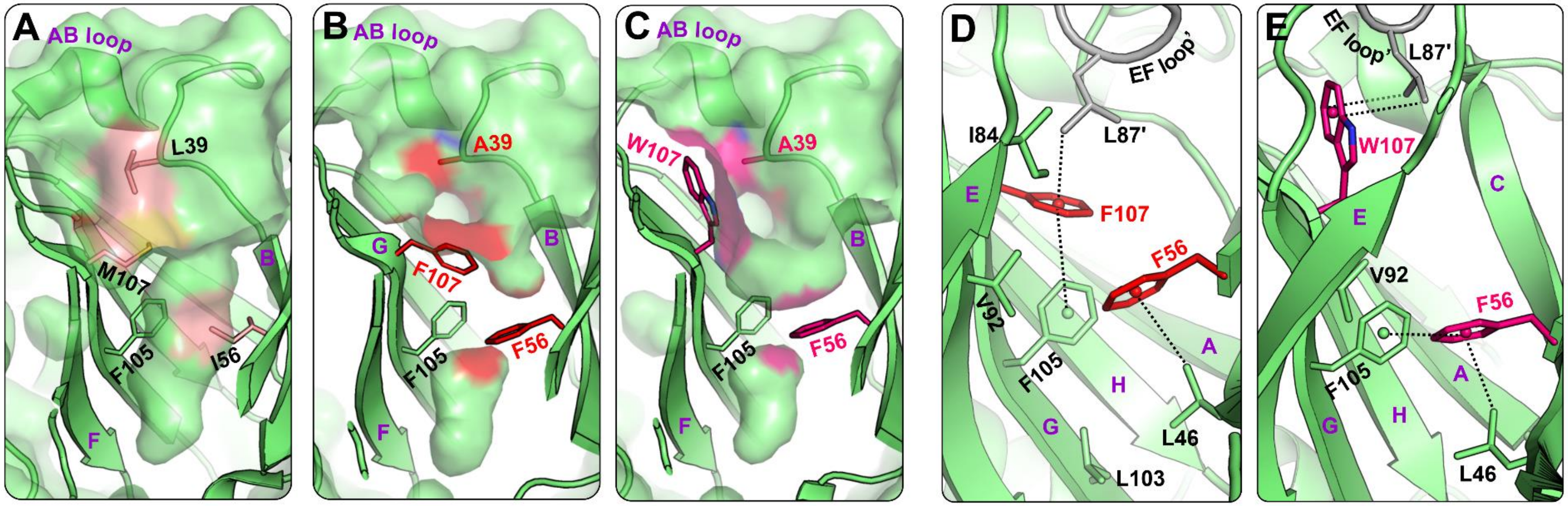

Fig. S3. Structures of unliganded WT BLG and mutants FAF and FAW. The shape of the binding pocket inside the β-barrel in (A) WT lactoglobulin (PDB: 6QI6), (B) FAF, and (C) FAW mutant. The molecular surface was drawn using a probe radius of 1.4 Å. Contribution of residues at positions 39, 56 and 107 to the molecular surface is marked by pink (panel A, yellow for sulfur), red (panel B, blue for nitrogen) and dark pink (panel C, blue for nitrogen). Network of interactions stabilizing the aromatic residues in the modified β-barrel of variant (D) FAF and (E) FAW. EF loop from a symmetry-related molecule (EF loop') is marked in gray. Selected CH···π interactions are marked with black dashed line to ring centers marked by small spheres.

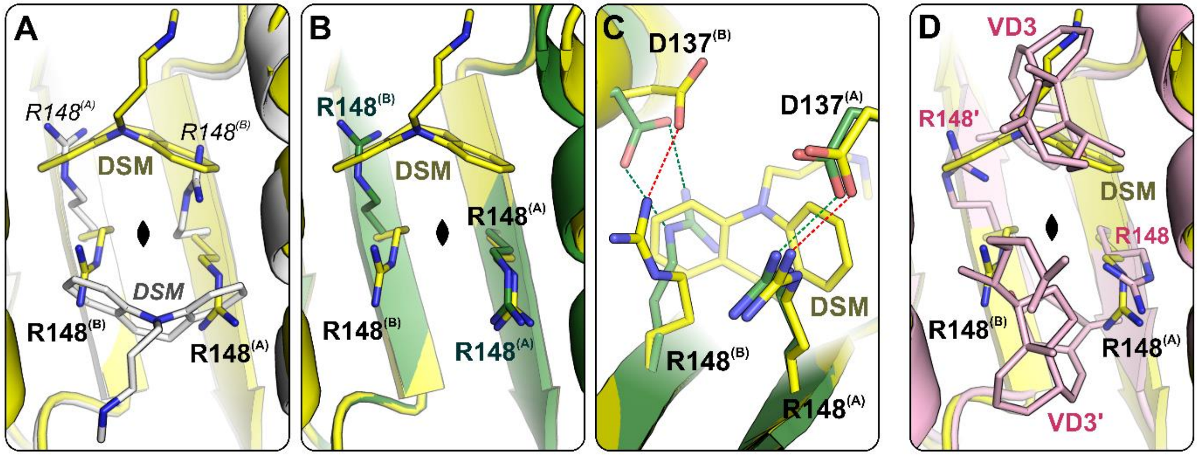

Fig. S4. Binding of ligand at BLG dimer interface. (A) Distortion of the twofold symmetry at the dimer interface: the FAF-DSM structure (yellow, bold) has been superposed on itself by 180° rotation (light grey, italics). The superposition shows that the symmetrical image of the cavity occupied by DSM is blocked by Arg148. (B) Superposition of unliganded FAF (dark green) and FAF-DSM complex (yellow). (C) Structural changes around Arg148 associated with DSM binding at the dimer interface in the FAF-DSM complex (yellow) in relation to unliganded FAF (dark green); green dashed lines mark H-bonds in unliganded FAF, while red dashed lines mark H-bonds in the FAF-DSM complex. (D) Superposition of the FAF-DSM structure (yellow) on the structure of the BLG-VD3 complex (PDB: 2GJ5, pink). In the BLG-VD3 structure the twofold crystallographic symmetry is preserved and two ligand molecules, VD3 and its symmetry mate VD3', are present at the dimer interface. The molecular dyad is represented by a black lens symbol.

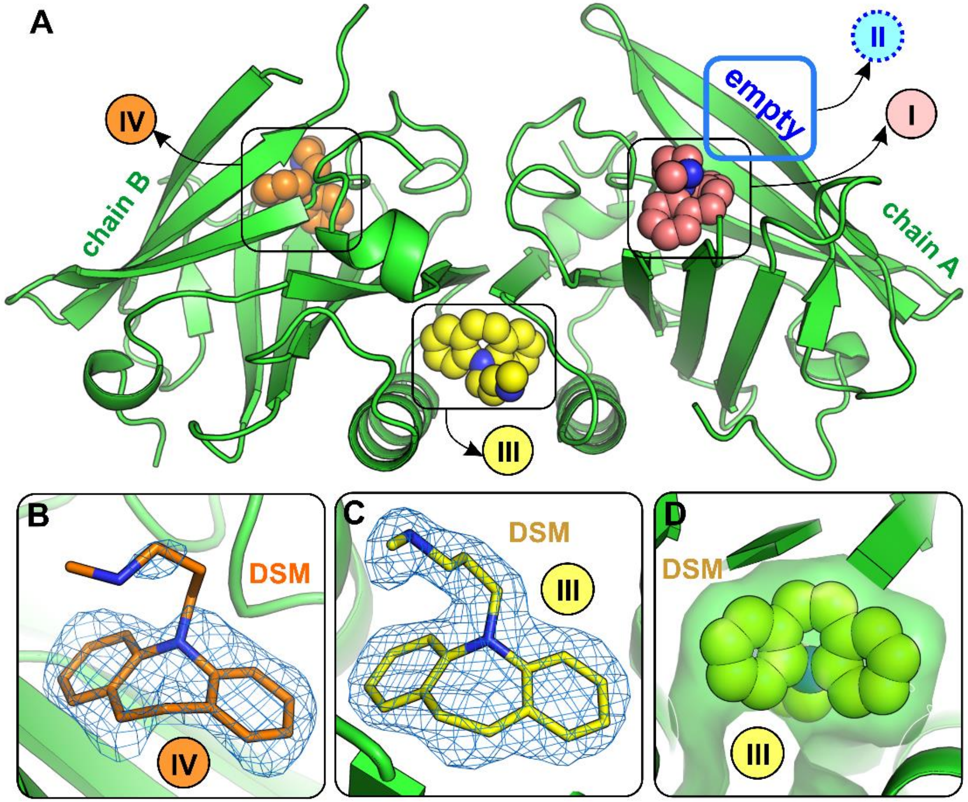

Fig. S5. Crystal structure of the FAW-DSM#2 complex. Ligand molecules were found inside the β-barrel (I and IV) and at the dimer interface (III). No DSM II molecule was bound at the β-barrel entrance (empty site II). (B-C) 2Fo-Fc electron density map contoured at 1.0σ around ligand molecules IV and III (difference omit maps are presented in Fig. S1). (D) Selectivity of the binding site III at the dimer interface is determined by perfect shape complementarity between the ligand and binding cavity.

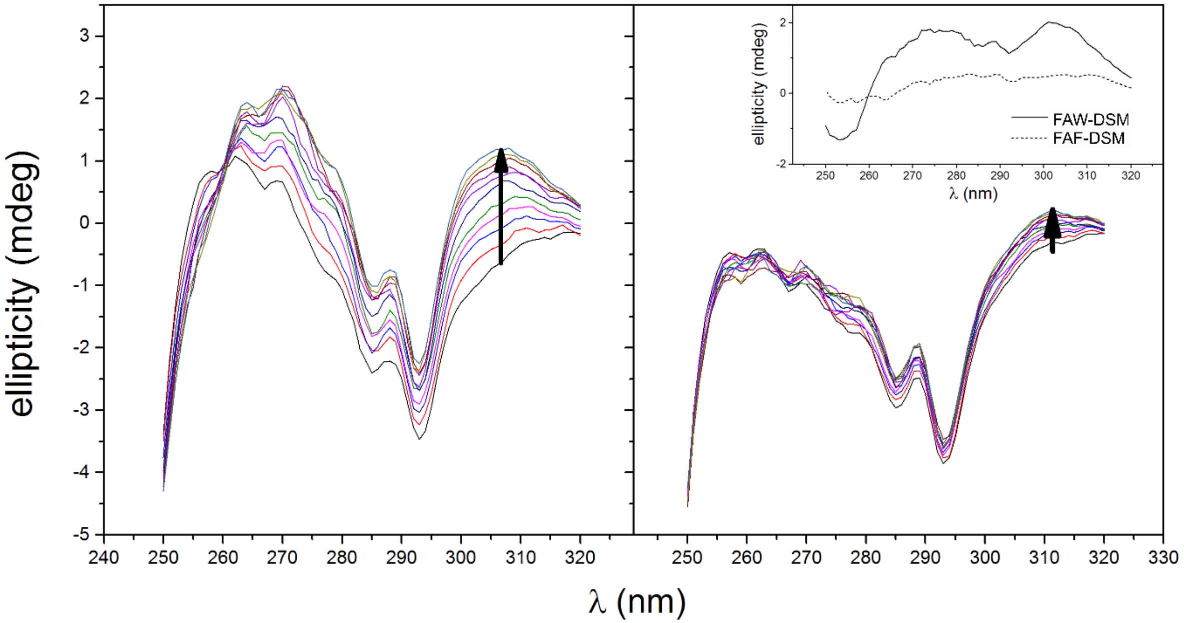

Fig. S6. CD spectra recorded during titration of FAW (left panel) and FAF (right panel) with DSM in phosphate buffer pH 7.5. The ligand as added as 10 mM stock solution in DMSO into 30 μM protein solution until threefold molar excess was reached. The arrows indicate the direction of the signal changes during DSM titration. Inset: induced CD spectra of DSM in complexes with FAW and FAF in molar ratio 3:1.

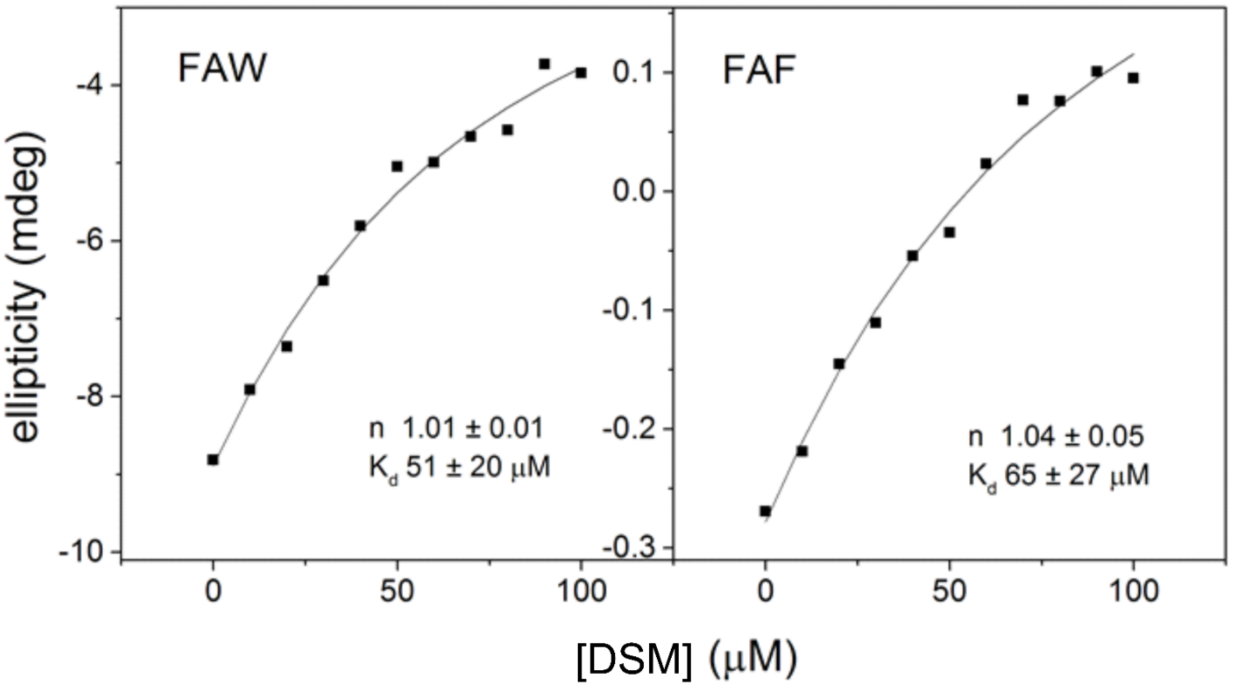

Fig. S7. Titrations of DSM into FAW and FAF solutions monitored by ICD in 50 mM phosphate buffer pH 7.5 at room temperature. The ligand was added as 10 mM stock solution in DMSO into 30 μM protein solution. The solid lines present the best fit of the data to the model of one set of binding sites.

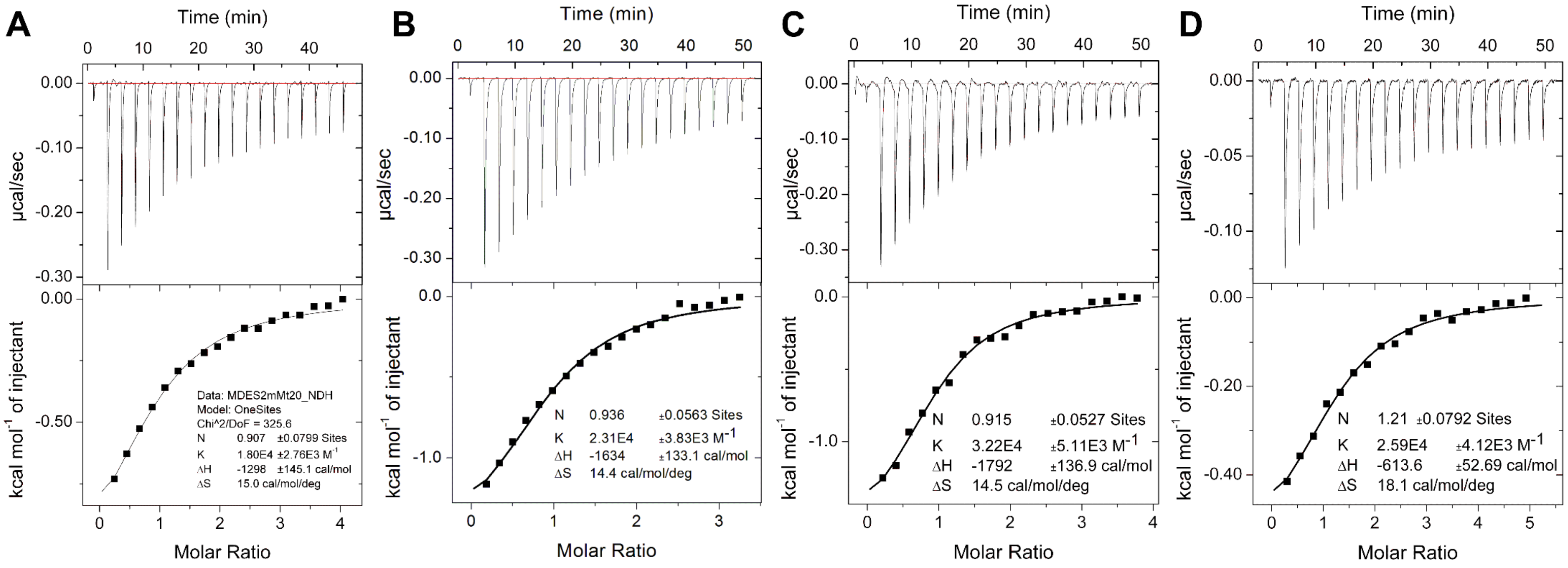

Fig. S8. The results of BLG titration with DSM. Sample raw data (upper panels) and binding curves obtained after their integration (bottom panels) with the best fit of one set of binding sites model obtained from ITC titrations at pH 7.5 of (A) 95 µM of native protein (B) 127 µM of FAF mutant and (C) 100 µM of FAW mutant in the cell with 19 of 2 µl injections of 2 mM, 1.83 mM, and 2 mM of DSM, respectively. (D) ITC titration of 79 µM FAF mutant with 2 mM of DSM at pH 8.5.

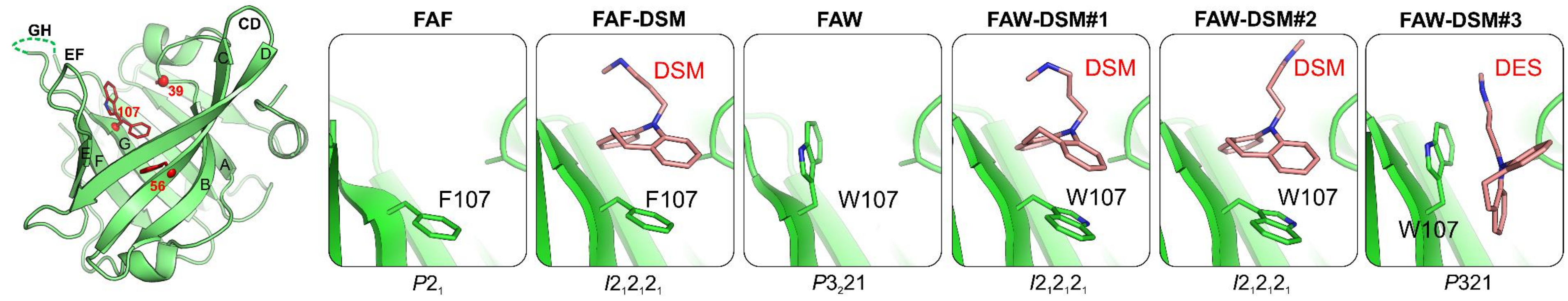

Fig. S9. The cartoon drawing on the left shows the structure of the BLG protomer with the mutation sites: L39A, I56F and M107F (or M107W) marked by red spheres or sticks; residue 107 (Phe107 in FAF or Trp107 in FAW) has different conformation in unliganded FAF and FAW. Panels in frames: Conformations of residue 107 (Phe or Trp) and position of ligand in the β-barrel in the crystal structure of the FAF-DSM and FAW-DSM complexes and unliganded proteins. In the FAW-DSM#1 and FAW-DSM#2 complex, the electron density shows two possible conformations of Phe107, however in the final models deposited to the PDB (ID: 7Q2O and 7Q2P), only one the major occupancy conformer (Table S3) was modelled.

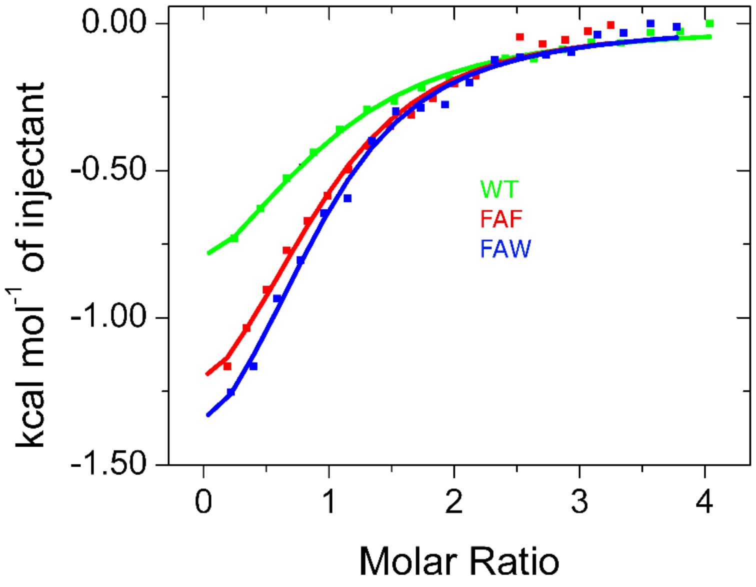

Fig. S10. Superposition of binding curves obtained after integration of sample raw data with the best fit of one set of binding sites model obtained from ITC titrations of 95 µM native protein (green), 127 µM FAF mutant (red) and 100 µM FAW mutant (blue) in the cell with 19 µl injections of 2 mM, 1.83 mM, and 2 mM of DSM at pH 7.5.

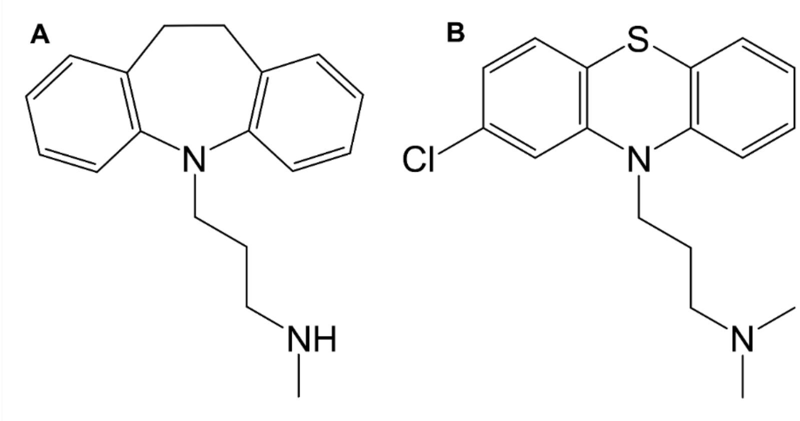

Fig. S11. Chemical structures of (A) desipramine and (B) chlorpromazine.

Supplementary References

Agudelo, D., Beauregard, M., Bérubé, G. & Tajmir-Riahi, H. A. (2012). *J. Photochem. Photobiol. B Biol.* **117**, 185–192.

Al-Shabib, N. A., Khan, J. M., Malik, A., Alsenaidy, M. A., Rehman, M. T., AlAjmi, M. F., Alsenaidy, A. M., Husain, F. M. & Khan, R. H. (2018). *J. Mol. Liq.* **269**, 511–520.

Birch, J., Khan, S., Madsen, M., Kjeldsen, C., Møller, M. S., Stender, E. G. P., Peters, G. H. J., Duus, J., Kragelund, B. B. & Svensson, B. (2021). *ACS Omega*. **6**, 9039–9052.

Collini, M., D'Alfonso, L., Molinari, H., Ragona, L., Catalano, M. & Baldini, G. (2003). *Protein Sci.* **12**, 1596–1603.

Ghalandari, B., Divsalar, A., Saboury, A. A., Haertlé, T., Parivar, K., Bazl, R., Eslami-Moghadam, M. & Amanlou, M. (2014). *Spectrochim. Acta - Part A Mol. Biomol. Spectrosc.* **118**, 1038–1046.

Labra-Núñez, A., Cofas-Vargas, L. F., Gutiérrez-Magdaleno, G., Gómez-Velasco, H., Rodríguez-Hernández, A., Rodríguez-Romero, A. & García-Hernández, E. (2021). *Arch. Biochem. Biophys.* **699**, 108750.

Liu, J., Jiang, L., Zhang, Y., Du, Z., Qiu, X., Kong, L. & Zhang, H. (2017). *RSC Adv.* **7**, 45960–45967.

Loch, J. I., Bonarek, P., Siuda, M., Wróbel, P. & Lewiński, K. (2021). *Acta Biochim. Pol.* **68**, 23–28.

Loch, J. I., Bonarek, P., Tworzydło, M., Polit, A., Hawro, B., Łach, A., Ludwin, E. & Lewiński, K. (2016). *Mol. Biotechnol.* **58**, 605–618.

Lübke, M., Guichard, E., Tromelin, A. & Le Quéré, J. L. (2002). *J. Agric. Food Chem.* **50**, 7094–7099.

Maity, S., Pal, S., Sardar, S., Sepay, N., Parvej, H., Chakraborty, J. & Chandra Halder, U. (2016). *RSC Adv.* **6**, 112175–112183.

Pantusa, M., Bartucci, R. & Rizzuti, B. (2014). *J. Agric. Food Chem.* **62**, 4384–4391.

Stender, E. G. P., Birch, J., Kjeldsen, C., Nielsen, L. D., Duus, J. O., Kragelund, B. B. & Svensson, B. (2019). *ACS Omega*. **4**, 6165–6174.

Xu, H., Lu, Y., Zhang, T., Liu, K., Liu, L., He, Z., Xu, B. & Wu, X. (2019). *Food Chem.* **281**, 28–35.

Yang, M. C., Guan, H. H., Liu, M. Y., Lin, Y. H., Yang, J. M., Chen, W. L., Chen, C. J. & Mao, S. J. T. (2008). *Proteins Struct. Funct. Genet.* **71**, 1197–1210.
